# Purulent Ophthalmia a Source of Gonococcus Joint Disease in Infants

**Published:** 1899-01-28

**Authors:** 


### HOSPITAL CLINICS AND MEDICAL PROGRESS.

### PURULENT OPHTHALMIA A SOURCE OF GONOCOCCUS JOINT DISEASE IN INFANTS.

THE wanderings of the gonococcus are matters of considerable interest, illustrating as they do the tendency of a disease which depends on a microbial infection to take very different forms, and to occur in very different places, according to variations in original intensity, in individual resistance, and in a multitude of other so-called "accidental circumstances." The history of gonorrhœal rheumatism is a curious one. Clinically the connection between the primary disease and the occurrence of joint affections has always been observed, and as, in the parlance of days gone by, all these affections of the joints were called rheumatic, the term gonorrhœal rheumatism became part of the language of medicine. It seems clear, however, that there is a great gulf between this disease and rheumatism proper, and although some—Mr. Hutchinson for example—would maintain that diathesis has much to do with deciding that a given case should show arthritic manifestations, other explanations than rheumatism have been sought for the occurrence of the joint affections which are found as complications of gonorrhœa. More especially has it been maintained on the one hand that the disease is a form of pyæmia, and on the other that it is due to some more or less occult action of the nervous system, that easy going *deus ex machinâ*, which seems to some a fitting and sufficient explanation of all pathological difficulties. Perhaps a still newer explanation is that the disease is a "toxic" manifestation, toxins again being almost as useful as "reflex action" in affording comfort to puzzled minds. Latterly, however, the knowledge that gonorrhœa is due to the local development of the gonococcus has led to a search for this microbe in those maladies which clinical experience has shown to be common accompaniments of the disease; and it appears to have been satisfactorily proved that in at least a certain proportion of the cases of gonorrhœal rheumatism, or, as it would now seem more proper to call it, gonorrhœal synovitis or arthritis, the joint inflammation is due to the same cause as the original discharge, namely, the gonococcus. This being granted there is no difficulty in accepting the proposition put forward by Mr. Clement Lucas at the meeting of the Royal Medical and Chirurgical Society last Tuesday to the effect that purulent ophthalmia, being mostly of gonorrhœal origin, may set up a secondary gonococcus joint disease. He had drawn attention in the year 1885 to a form of joint disease in infants, which before that time had not been recognised or described, in regard to which he had then said: "I am not aware that any connection between ophthalmia neonatorum and synovitis has ever been observed or described; but there seems no just reason if, as is generally supposed, the synovitis of gonorrhœa is the result of absorption of morbid products from the urethral mucous membrane, why the conjunctival mucous membrane should not offer an equally favourable absorbing surface. It is scarcely probable that the inflammation of these two joints could be referred to any other cause; and in my own mind there exists no doubt whatever that this is a case of gonorrhœal rheumatism, consequent on absorption from the conjunctival surface." Substitute the word "gonococci" for "morbid products" and we have the theory which Mr. Clement Lucas now put forward, supporting his thesis by 23 cases collected from various sources. Of the 23 cases referred to, 18 were due to ophthalmia neonatorum caused by inoculation from mothers who were suffering from vaginal discharges at the timeof labour. The remaining five had the conjunctivæ inoculated at a subsequent period.

The third day was that on which the purulent ophthalmia most generally declared itself, and the date, after the appearance of the ophthalmia, at which the joint disease might be expected to declare itself was towards the end of the second week or during the third week. Another point worth noting is that the disease showed a very marked predeclition in favour of the knees, which were inflamed in no less than 14 cases. Most of the other larger joints, however, have been found to be affected, and, curiously, the knee and wrist on the left side were attacked more frequently than those on the right. This is explained by the fact that mothers and nurses usually by preference carry the infant on the left arm, so as to have their own right hand more free for use, so that the infant's left knee and left wrist are the most exposed to cold and injury, and thus become affected with the gonococcus just in the same manner as, in the well-known experiments on the lower animals, a large proportion of sprained joints become tuberculous if the animal be injected with the tubercle bacillus in some other part of the body. The real nature of the disease appears to have been demonstrated by Deutschmann, in 1890, who not only showed the gonococcus in the secretion of the conjunctiva in these cases of ophthalmia neonatorum, but, by aspirating an inflamed knee of an infant three weeks old suffering from purulent ophthalmia, was able to show the gonococcus in the secretion of the joint. Mr. Clement Lucas, however, struck by the fact that gonorrhœal rheumatism rarely suppurates, while three of the 23 cases reported had ended in suppuration, suggested that in some of the cases there might be a double infection—a gonococcus inflammation *plus* the inflammation due to some other microbe.

In regard to the course and duration of the disease, it was pointed out that the joint affection being secondary to a purulent infection of the conjunctival mucous membranes, the course of the disease would bear a definite relation to the course of the disease at the infecting source. It was stated, however, that the acuteness of the ophthalmia did not appear to bear any relation to that of the joint disease, and seemed to depend more on the state of resistance on the part of the infant. Although some few of the cases had recovered in from ten days to a fortnight, the majority ran a course of from three to five weeks before complete resolution took place. Complete resolution might, however, be always looked forward to with confidence, even when the inflammation was so intense as to suggest suppuration.

Although in the discussion the main thesis was generally sustained, exception was taken to many points, more especially to the statement that the treatment should be mainly directed to the early and complete cure of the purulent ophthalmia, which is the infecting source, it being pointed out that it is almost universally the case that a microbial infection which has once become generalised may continue its course quite independent of what happens to the original source of infection.

